# Clinical decision support system for child and adolescent mental health services: a formative usability study

**DOI:** 10.1186/s12911-026-03720-w

**Published:** 2026-07-20

**Authors:** Carolyn E. Clausen, Dipendra Pant, Roman Koposov, Øystein Nytrø, Bennett Leventhal, Stian Lydersen, Odd Sverre Westbye, Thomas Brox Røst, Norbert Skokauskas

**Affiliations:** 1 Regional Centre for Child and Youth Mental Health and Child Welfare (RKBU)-Central Norway, Department of Mental Health, Faculty of Medicine and Health Sciences, Norwegian University of Science and Technology (NTNU), Trondheim, Norway; 2 Department of Computer Science, Norwegian University of Science and Technology (NTNU), Trondheim, Norway; 3 Regional Centre for Child and Youth Mental Health and Child Welfare (RKBU)- Northern Norway, UiT The Arctic University of Norway, Tromsø, Norway; 4 Department of Computer Science, UiT The Arctic University of Norway, Tromsø, Norway; 5 The University of Chicago, Chicago, IL USA; 6 Department of Child and Adolescent Psychiatry, St. Olav’s University Hospital, Trondheim, Norway

**Keywords:** Child and Adolescent Mental Health Services (CAMHS), Clinical decision support system, User-centered design, Formative usability, Think-aloud, Human computer interaction

## Abstract

**Background:**

Child and adolescent mental disorders are creating significant demands on Norway’s general and specialized health services. Addressing these demands requires innovative approaches to increase workforce numbers and effectiveness. The Individualized Digital Decision Assist System (IDDEAS) is a Clinical Decision Support System (CDSS) for child and adolescent mental health services in Norway. IDDEAS 1.0 is a guidelines-based prototype with attention-deficit/hyperactivity disorder (ADHD) as the first model clinical paradigm. IDDEAS 1.0 is the focus of a formative usability study to examine clinicians’ information processing needs and their perceptions of the acceptability, functionality and ease of interactions with user interface (UI) and user-experience (UX).

**Methods:**

In collaboration with the Norwegian Association for Child and Adolescent Mental Health (N-BUP) a provisional list of practitioners in child and adolescent mental health services (CAMHS) was compiled for recruitment of potential participants. Child and adolescent psychologists and psychiatrists (*n* = 68) completed IDDEAS formative usability testing. Study participants also completed a think-aloud protocol in which they verbalized their thoughts about the experience in real time while assessing hypothetical patient case vignettes. By observing these representative, potential IDDEAS users, developers gain insight from their interactions with the prototype. A self-developed display and functional ease-of-use questionnaire, as well as the IDDEAS modified system usability scale and user engagement scales, were completed. All qualitative data were analyzed using both protocol analysis and content analysis.

**Results:**

Examination of the formative usability identified a participant appreciation of the side-by-side guideline/patient view, simplistic UI presentation, and alignment with their diagnostic routines. 89% of usability comments informed next-step improvements pinpointing forced linear navigation, ADHD focused prototype, and ambiguous prompts. This resulted in proposed enhancements centered on dynamic criteria selection, optional guideline choice, individualized content, and seamless electronic health records integration.

**Conclusion:**

The scenario-based exploration of the IDDEAS 1.0 prototype allowed CAMHS clinicians to offer honest reflections about receiving decision-support, and what they might potentially need for such support to enhance their clinical decision-making and information processing at the point of care. Functional adjustments in IDDEAS were recommended, with a focus on workflow cohesion and individualized adaptability for optimal ease of use and personalized patient care.

**Supplementary Information:**

The online version contains supplementary material available at https://doi.org/10.1186/s12911-026-03720-w.

## Introduction

Child and adolescent mental disorders are common and often debilitating, and without appropriate and timely interventions, children and adolescents with mental disorders can face lifelong challenges [1–3]. Copeland et al. reported that untreated childhood mental disorders can lead to worsened health, financial instabilities, and impaired social functioning in adulthood [4]. In Norway, increasing numbers of adolescents have self-reported mental health symptoms over the past decade [5–7]. Following the COVID-19 pandemic, in 2021 the country’s Child and Adolescent Mental Health Services (CAMHS) faced a 15.7% increase in patients aged 13–15 years old receiving care through a consultation with a specialist and 30% increase in hospitalizations [8]. A 2022 National Patient Registry (NPR) study found that after the pandemic the number of 13- to 15-year-olds receiving a CAMHS consultation for Attention-Deficit/Hyperactivity Disorder (ADHD) increased by 21.6%, and by nearly 9% among 6–12-year-olds [8]. According to the Escalation Plan for Mental Health (2023–2033), while the goal is to reduce the wait time for specialist child and adolescent mental healthcare to an average of less than 35 days [5], CAMHS remains overburdened, leading to long wait times and delayed access to essential assessments and treatments [5, 6, 8–11].

Advanced health information technologies (HIT), such as clinical decision support systems (CDSSs), have the potential to optimize the use of existing resources, thereby increasing service capacity in a cost-effective and efficient manner [12, 13]. CDSSs have been successfully implemented for various care services, such as imaging support [14], diagnostics support [15], and to assist with quality assurance of clinical management [13, 16, 17]. While CDSS for general medicine has been broadly studied, research on CDSSs for mental healthcare remains limited in comparison [18–20]. CDSSs within mental healthcare target a variety of specific mental health problems and/or disorders [19, 20], such as depression [21–25], schizophrenia and other psychotic disorders [26–29], and substance use disorder [30, 31]. Most mental health CDSSs concern adult mental health, with a small number for child and adolescent mental health, which are predominantly in primary care settings, not CAMHS [20, 32].

Depending on its design, a CDSS can improve the precision and timeliness of services by providing targeted clinical knowledge and evidence-based suggestions to clinicians in real time [13, 33, 34]. CDSSs incorporate clinical practice guidelines and/or aggregated patient health record data, commonly referred to as “big data,” to assist clinicians in making informed decisions [32, 35–37]. Predictive algorithms and Artificial Intelligence (AI) models can analyze extensive historical data to generate predictions about potential future outcomes [38, 39]. Predominantly AI-enabled CDSSs (AI-CDSS) utilize statistical task-specific models developed using supervised Machine Learning (ML) techniques [40], commonly applied within diagnostic imaging and risk prediction [40–42]. While AI has shown potential to enhance diagnostic accuracy [43], efficiency, and personalization of assessments for complex neurological and mental health disorders [43–48], the integration of AI-CDSS in clinical practices remains limited [49]. Despite AI-CDSS intent to use AI to enhance support provided at the point of care to further enhance evidence-based and personalized mental healthcare, there are still ethical concerns with regards to data sensitivity and inappropriate generated recommendations based on data biases [44, 46, 50–52]. Advancing the potential for AI-CDSS in mental healthcare, including CAMHS, will rely on close human-AI collaboration, requiring thorough user-oriented CDSS design and development research [18, 19, 48].

Optimally, CDSS development must consider user-centered design (UCD), aspects of human-computer interaction (HCI), and overall user-experience (UX) [20, 53, 54]. There are several key factors that influence UX, including the system’s usefulness and usability [55, 56]. It is generally accepted that CDSS system development should utilize formative usability testing [54, 57, 58] to establish a robust contextual understanding of a system’s usability and users’ needs, clearly define the clinical problem(s) to be addressed [59], and determine an effective strategy for integrating clinical decision support [59–62]. Usability testing commonly involves moderating techniques (i.e., think-aloud (TA)) and/or surveying, such as the completion of the System Usability Scale (SUS) [63–66]. Scenario or simulation-based testing, as well as workflow analysis, are also conducted to assess usability at the earliest stages of system development as they are important to UCD and the application of methods to ensure that the final CDSS design is consistent with professional clinical decision-making (CDM) and overall workflow integration [53, 67–70].

While CDSSs exist in other areas of medicine in Norway, such as one system designed to identify potential allergic reactions with anesthesia in hospitals [71], or one developed for the management of musculoskeletal pain disorders in primary care [72], a CDSS has yet to be developed for CAMHS [12, 20, 73, 74]. The Individualized Digital Decision Assist System (IDDEAS) project is developing a guideline-based CDSS, that leverages big-data and integrates Explainable Artificial Intelligence (XAI) and interpretable ML techniques, to deliver transparent decision support to Norwegian CAMHS clinicians in real-time, at the point of care. IDDEAS is a multiphase UCD based CDSS development research project, with the aim to implement a system that can optimally improve the quality of care with enhanced efficiency by providing support to CAMHS practitioners through the diagnosis and treatment of patients [75, 76].

The present study explores the perspectives of Norwegian CAMHS clinicians on the usability of the IDDEAS prototype 1.0, with a focus on HCI factors relevant to further system development [75]. To set a base for further system development, the formative usability testing of prototype 1.0 uses ADHD as the first clinical paradigm [35, 75, 77], as it is one of the most common developmental disorders among children, with 3.8% of all Norwegian children diagnosed with ADHD by 12 years of age [78], yet considerable clinical variation in the diagnosis and treatment exists between CAMHS clinics [78–81]. Additional specific study aims explore how clinicians engage in clinical reasoning and identify key components of clinical workflows that will benefit from enhanced decision support, while also obtaining a detailed understanding of the perceived usability and clinical relevance of the IDDEAS 1.0 prototype.

## Methods

### Participants

#### Selection criteria and sampling methods

Eligible study participants were either practicing Norwegian child and adolescent psychologists or psychiatrists, the only clinicians in Norway authorized to make a clinical diagnosis of mental disorder and prescribe treatment. To help with participant recruitment N-BUP (Norwegian Association for Child and Adolescent Mental Health) provided a list of child and adolescent mental health clinicians to serve as potential participants. Additional potential participants were also suggested by BUP clinic representatives and local clinical experts working in collaboration with IDDEAS. A total of 208 psychologists and psychiatrists were contacted and invited to participate. Ultimately, 68 clinicians accepted invitations and participated in assessment of the IDDEAS prototype.

#### Sample size

Necessary sample size was estimated based on the assumption that the effect of the decision support system will increase specificity from 0.50 to 0.80, and only moderately change sensitivity. If the two results from the same clinician were independent, a sample size of 39 clinicians is sufficient to obtain 80% power with significance level *p* = 0.05. However, decisions from the same clinician are positively dependent, leading to the estimate of a 30% increase in participants. This suggests a sample size of 51 clinicians to maintain the same power. This component of the study falls outside of the scope of this article and will be reported separately, as with IDDEAS development and subsequent testing findings.

The first 20 participants were included as the sample for the TA data to avoid qualitative data oversaturation and allow for comparative analyses with parallel semi-structured interviews [77]. In accordance with other TA studies and qualitative research recommendations, data saturation was determined based on category information redundancy (i.e., Stopping Criterion) and code frequency saturation [82] for both the protocol analysis and content analysis [82, 83].

### Materials and equipment

#### IDDEAS 1.0

The IDDEAS project is a mixed-methods study to evaluate the IDDEAS CDSS for diagnosis and treatment of child and adolescent mental health in Norwegian CAMHS [75]. The project is organized into four stages: (1) Identification of needs and IDDEAS preparations; (2) The IDDEAS prototype design and evaluation; (3) Prototype refinement and iterative evaluation(s); (4) IDDEAS implementation and dissemination [75, 77]. Additional contributions to the IDDEAS development are published separately [35, 77, 84–86]. IDDEAS 1.0 is a guideline-based CDSS prototype developed by the IDDEAS team, an international team of Information and Communication Technology (ICT) and health professionals. IDDEAS 1.0 uses ADHD as the first clinical paradigm/case example, with its prevalence in practice and practitioner exposure can serve as an informative base for further system development. The prototype is an interactive platform that provides the clinician with access to rule-based, generated recommendations from clinical guidelines to serve as decision support and aid through diagnostic assessment and treatment planning/management procedures. Previously validated IDDEAS clinical ADHD guidelines [75] were modeled as a “decision tree” and implemented within the IDDEAS 1.0. The “decision tree” format allows for participants to select whether the patient fulfills assessment criteria and based on their selection, generates and presents the next branch of criteria options to assess for that fall in line with the specific clinical guideline recommendations [77].

An initial focus group evaluation was conducted with IDDEAS consortium members, which informed revisions to the prototype’s format. The IDDEAS consortium is an international multidisciplinary team, including researchers, developers, practitioners, and health informatics specialists, who provide collaborative efforts and support for the IDDEAS team, who are directly responsible for the research and management of the project. The IDDEAS platform was then implemented within a web interface and pre-tested by an initial child and adolescent clinician focus group (*n* = 4) consisting of Norwegian CAMHS clinicians (two psychologists and psychiatrists) who were not part of the IDDEAS group.

#### Patient case scenarios

**Fig. 1:**
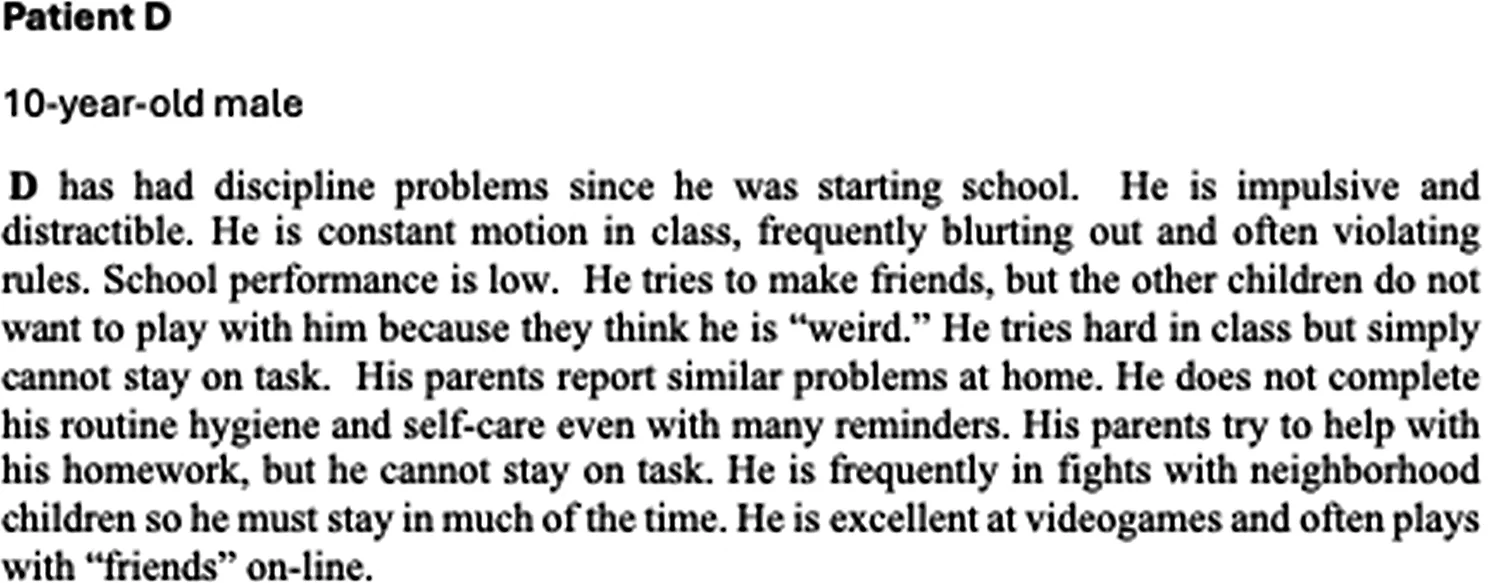
Example patient case scenario

The IDDEAS team’s child and adolescent mental health clinical experts (NS, BL, RK, and OSW) collaboratively developed 20 patient case scenarios to serve as hypothetical patients referred to CAMHS. Prior to finalization, the cases were validated in collaboration with IDDEAS consortium members who serve as clinical practitioners in the field. The case scenarios were intentionally designed to vary in complexity and amount of patient information provided (see Fig. 1).

#### Questionnaires

Participants were asked to complete four instruments: a demographic questionnaire, the IDDEAS Display Functionality and Ease-of-Use questionnaire, the adapted System Usability Scale (SUS) [65, 87], and the adapted User Engagement Scale (UES) [88] (see Table 1). The demographic questionnaire was a self-developed scale, internally reviewed in both English and Norwegian. The questionnaire included items on participants’ identified gender, clinical role, and duration of work experience with CAMHS. The IDDEAS Display and Function- Ease-of-Use questionnaire consists of 10 items to which participants can respond using a 5-item Likert scale (see Supplementary File 1). The scale was developed specifically for this study, designed to assess participant perception of the helpfulness and potential usefulness of specific IDDEAS 1.0 functions, as well as of the displayed attributes of the user interface (UI). Following the formal translation and cultural adaptation procedures, both the SUS [87, 89, 90] and the UES [88, 91–93] were employed in the study. For both questionnaires we conducted forward translation, back translation, and validation evaluations to assess the translation [94–96].

**Table 1:** IDDEAS prototype 1.0 formative usability testing questionnaires

Title of Questionnaire	Abbreviation	About (Scale Type, No. of Items)	Measure	Reference
System Usability Scale	SUS	Translated and adapted version of scale for the IDDEAS context (Likert scale, 10 items)	Subjective usability of a system	[87]
User Engagement Scale	UES	Translated and adapted version of scale for the IDDEAS context (Likert scale, 30 items)	Subjective user engagement (Focused Attention (FA), Perceived Usability (PU), Aesthetic Appeal (AE), and Reward (RW)	[88]
IDDEAS Display and Function Ease-of-use	N/A	Self-developed scale (Likert scale, 10 items)	IDDEAS display/functions perceived helpfulness, appropriateness and ease-of-use	N/A
IDDEAS Demographic Questionnaire	N/A	Self-developed scale (nominal/ordinal, 3 items)	Foundational social and demographic characteristics of participants	N/A

Confirmatory Factor Analyses (CFA) were conducted to evaluate the factor structures of the translated and culturally adapted versions of the SUS and UES. The Weighted Least Square Mean and Variance (WLSMV) estimator was applied to account for ordinal categorical data. A one-factor structure (i.e., Usability) and a two-factor structure (i.e., Usability and Learnability) were analyzed for the SUS [65, 89, 97], and a four-factor structure was analyzed for the UES: Focused Attention (FA), Perceived Usability (PU), Aesthetic Appeal (AE), Reward (RW) [88, 98]. The factor structures were based on the original, previously validated scale [88, 91, 93, 99]. The goodness of fit indices applied included the Root Mean Square Error of Approximation (RMSEA), Comparative Fit Index (CFI), and Tucker Lewis Index (TLI). A RMSEA value of ≤ 0.80, CFI value and TLI value of ≥ 0.95, were considered to signify an adequate model fit [100–102]. The CFA analyses were conducted in Mplus 8, and the other analyses were conducted in SPSS version 29.

The CFA of the UES supported a second-order engagement factor with four latent factors of FA, PU, AE and RW. Overall, the factor loading for each latent variable indicated correlation amongst FA, PU, AE and RW. The UES model goodness of fit indices indicated a marginally good fit based on RMSEA = 0.092, and a good fit with TLI = 0.94 and CFI = 0.95. For the IDDEAS translated and culturally adapted SUS, the CFA neither fully supported a one-factor model nor a two-factor model. The one-factor model found a poor goodness-of-fit [RMSEA = 0.170, CFI = 0.940, TLI = 0.923] and standardized factor loadings ranged from 0.54 to 0.87. The CFA two-factor model also had a poor goodness of fit [RMSEA = 0.154, CFI = 0.952 and TLI = 0.937]. Standardized factor loadings ranged from 0.58 to 0.87. The graphical depictions of the SUS CFA one and two-factor model are both presented in Appendix I, with the graphical depiction of the UES hierarchical CFA presented in Appendix II.

### Procedure

The clinicians who expressed interest in participating in the study were sent the IDDEAS information package and invited via email to evaluate the IDDEAS 1.0 prototype. The package included an introduction to IDDEAS 1.0 and an overview of the evaluation procedure.

All IDDEAS 1.0 evaluation sessions were conducted by the first author (CC), either online or in person, based on participant preference. At the start of each session, participants provided informed consent for participation, prior to receiving access to the platform. Each participant completed a concurrent Think-Aloud (TA) method for CAMH patient case scenario assessment, both with and without the use of IDDEAS 1.0. The TA method is a user-centered method intended to elicit thought processes followed when completing a given task [83, 103, 104]. The allocation sequence of the patient case scenarios to be assessed was computer generated and known only to CC. For the completion of the TA method, the participants were asked to speak aloud describing their natural thought process and what came to mind while assessing the allocated patient case scenarios. At the end of the TA method, participants were then prompted to complete the questionnaires via the embedded WebForm in the online testing platform and a semi-structured interview about how they experienced using the IDDEAS prototype as well as their initial perceptions of its usability. The findings from the formative usability testing semi-structured interviews are reported separately [77].

### Data collection

All rating instruments were embedded within the IDDEAS testing platform, with responses automatically recorded and linked to the corresponding participant ID. All submitted data were saved on the encrypted WebForm platform. All data were anonymized for analyses by replacing participant identifying information with randomly selected participant codes that were kept separately and securely from the rest of the data. CC was responsible for management of the data files and direct access to the data.

All TA qualitative data gathered during the IDDEAS 1.0 evaluations were recorded and transcribed verbatim, based on two passes, to ensure that the transcripts accurately reflect the recordings and prevent errors or missing content. All data was only accessible by CC and stored securely on the Norwegian University of Science and Technology (NTNU) server.

### Data analysis

#### Questionnaires

For the IDDEAS Display Functionality and Ease-of-Use questionnaire, the distribution of responses was analyzed by calculating the percentage of participants selecting each response category. To assess the relevance and appropriateness of the system’s design features, the analyses specifically focused on evaluating the perceived clarity of the interface layout, ease of navigation, and overall user experience. For the IDDEAS modified SUS and UES, the mean (M) and standard deviation (*SD*) for the total SUS scores and total UES scores were calculated in accordance with previously established procedures and scoring standards [88, 89, 92, 98, 99, 105]. Cronbach’s alpha (*α*) was calculated for each scale as a measure of reliability, with the value of *α* between 0.7 and 0.9 considered to be optimal [93, 106–109]. Missing data were addressed following complete case analysis methods. Missingness due to the web platform questionnaire integration errors was considered missing completely at random (MCAR) and omitted.

#### Think-aloud method

Protocol analysis, and content analysis, were both applied to the TA data. We applied protocol analysis with the aim of finding out more about specific components of their CDM within clinical scenarios and the information processing involved in making complex clinical decisions along their cognitive pathway. Whereas the content analysis techniques were applied to thoroughly interpret the TA content concerning the CDSS itself to gain insight into how clinicians perceive usability of the CDSS at this stage and how these interplay with their workflow. After all TA data was transcribed verbatim, protocol analysis was applied to each transcript and consisted of three stages: Referring Phrase Analysis (RPA), Assertional Analysis (AA), and Script Analysis (SA) [83, 103]. For RPA, the TA data were broken into segments (i.e., sentences or clauses), from which all mentioned objects/noun phrases (i.e., patient or assessment tool) were then identified and defined as concepts and sub-concepts. For AA, all identified concepts were then assessed in relation to the rest of the information within the segment and defined as relational assertions. For SA, the reasoning processes (i.e., operators) were identified and then coded into categories that represent the main clinical reasoning and decision-making process [103]. From the preliminary analysis of transcripts, a protocol analysis provisional coding scheme was generated by CC and verified through discussion with coauthor NS. The provisional coding scheme was then also verified by the involved IDDEAS team coauthors and finalized by CC. For verification, clinical expertise was provided by BL, NS, OSW, and RK, who all have held clinical practitioner capacities in child and adolescent mental health. All IDDEAS team members were involved in verifying the final protocol analysis coding scheme. Each transcript was coded and analyzed in accordance with the final coding scheme to quantify the RPA, SA, and AA findings to inform cognitive pathway summary construction. For all the transcripts, the results from each analysis component were compiled to establish participant information-processing models. All information-processing models were compared to quantify identified commonalities and patterns in the participants’ CDM to establish a cognitive pathway summary [33, 83]. The pathway provides a general visualization of participants’ verbalized cognition as a sequence of internal states and information processing, intrinsic to the clinical workflow/pathway for patient assessment when interacting with the IDDEAS system.

For the content analysis, the data were deductively coded based on predetermined usability constructs derived from prior studies [57, 110] and inductively coded to account for the specific usability constructs that the TA content covered [111, 112]. Following inductive coding, all transcripts were thoroughly analyzed, and categories were revised to appropriately reflect the usability content in the data. A subsample of the transcripts was reviewed to establish potential coding categories before completing the analysis of all transcripts. The TA usability data were then further analyzed based on participants’ vocalized sentiment concerning any specific CDSS prototype attributes noted [111]. Each statement was categorized as either “critical” or “positive.” From the critical statements, any specified problems with the prototype were organized in accordance with the relevant usability construct (i.e., critique concerning a problem encountered with system navigation or clarity of content) [111, 113]. All noted problems were organized based on frequency of mention to acknowledge the relative severity of each perceived problem [114]. Any positively noted attributes or propositions for specific adjustments to be made to the IDDEAS prototype were also extracted [69]. All usability data were also coded, based on the respective User- System Interaction (USI) cycle stage in which the participant was engaged at the time [111, 113, 114].

## Results

Of the 208 clinicians contacted, upon completion of informed consent, 68 participated in the study, which is a 33% response rate (see Table 2). Data collection was predominantly conducted online from March 2021 through February 2023. Per participant preference and COVID-19 regulations at the time, six evaluation sessions were conducted in person, while 62 were held remotely.

**Table 2:** Participants’ social demographic characteristics

Social demographic characteristics	*n* = 68
**Gender**	
Male	26 (38%)
Female	41 (60%)
Non-binary*	1 (2%)
**Profession**	
Child and adolescent psychologist	55 (81%)
Child and adolescent psychiatrist	13 (19%)
**Duration of clinical experience in CAMHS**	
0–6 months	3 (4%)
6 months – 1 year	6 (9%)
1 year – 4 years	12 (18%)
More than 4 years	47 (69%)

*Non-binary in this case indicates the participant does not identify with either gender

### Quantitative findings

**Fig. 2:**
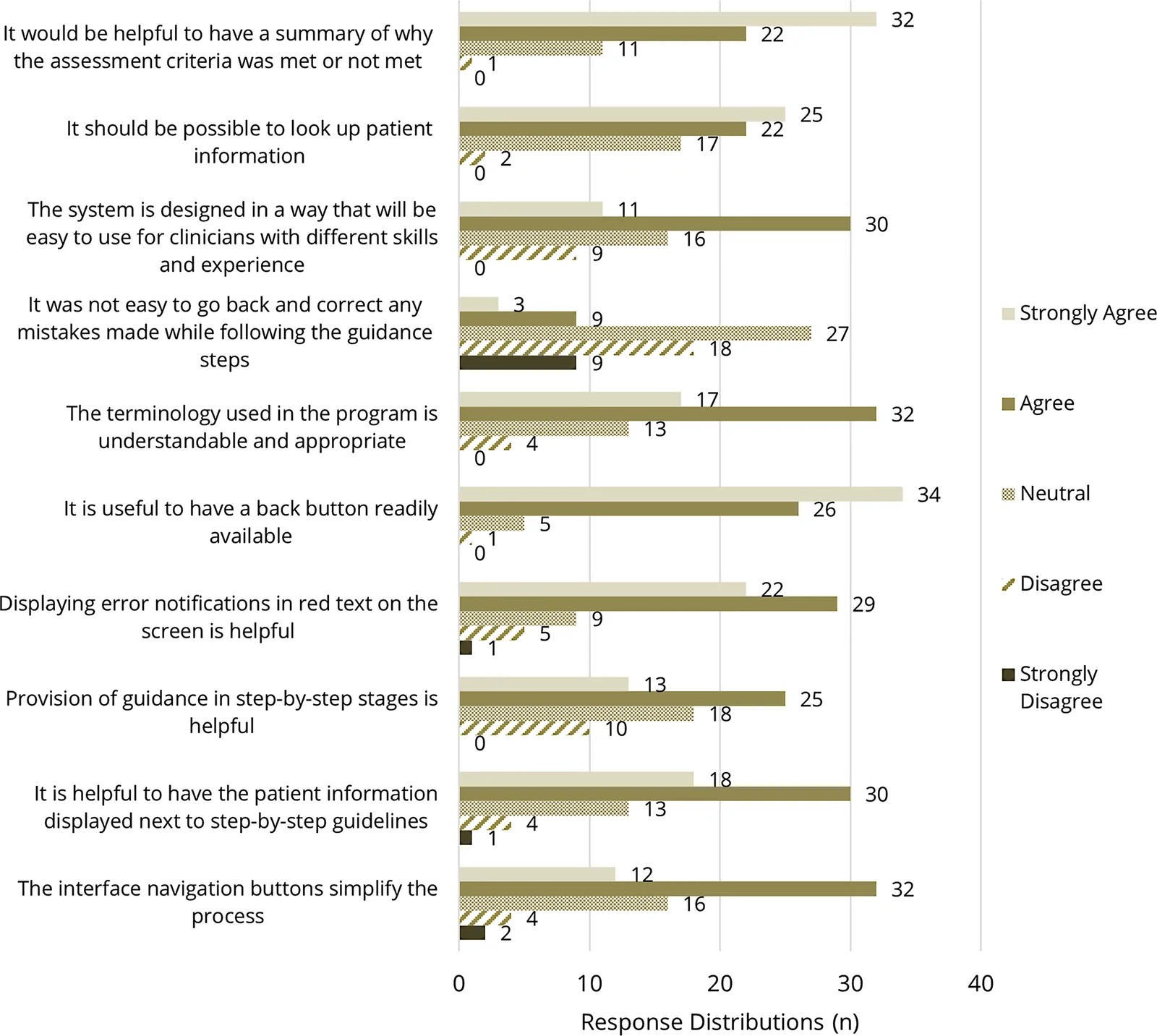
IDDEAS display and functional ease-of-use

Out of the 68 participants, 66 submitted a completed IDDEAS Display and Functionality Ease-of-Use questionnaire (see Fig. 2). Out of those 66 participants, 34 (52%) strongly agreed and 26 (40%) agreed with “*It is useful to have a back button readily available;”* 32 (48%) strongly agreed and 22 (33%) agreed with “*It would be helpful to have a summary of why the assessment criteria were met or not met;”* and, 25 (38%) strongly agreed, while 22 (33%) agreed with “*It should be possible to look up patient information.”*

Sixty-three participants completed the IDDEAS adapted and modified SUS. The IDDEAS SUS reliability analysis revealed a Cronbach’s α value of 0.86. The mean IDDEAS SUS score was 68.14 (*SD = 14.30*).

With 64 participants completing the IDDEAS adapted and modified UES, the overall average UES score was 13.49 (*SD = 0.37*). Among the four dimensions of user engagement, PU had a mean score of 3.74 (*SD = 0.65*), AE had a mean score of 3.49 (*SD = 0.69*), RW had a mean score of 3.39 (*SD = 0.73*), and FA had a mean score of 2.87 (*SD = 0.66*). The reliability analysis revealed all subscales of the modified UES – FA, PU, AE and RW– to have high internal consistency with α values of 0.81, 0.82, 0.86, and 0.91 respectively. The modified UES subscale scores and itemized response distributions are reported in Appendix I.

### Qualitative findings

#### Think-aloud: protocol analysis

The total duration of Think-Aloud (TA) sessions ranged from nine minutes to one hour and 33 min. For the TA data, a total of 2155 sentences were analyzed. RPA identified 10 main concepts and a total of four sub-concepts. For example, *Sign* and *Value* were main concepts without sub-concepts, whereas *Patient/Electronic Health Record (EHR)* had two sub-concepts: *Available Information* and *Missing/Needed Information*, as well as *Clinical Hypothesis* with two sub-concepts: *Diagnosis(es)* and *Related Factors*. Two of the main concepts were found to have two sub-concepts each. The finalized concepts/sub-concepts coding scheme serves as an ontology of the main clinical information and ideas on which the participants concentrate when assessing patient cases, while interacting with the IDDEAS prototype (see Table 3).

From AA, based on the deduced model, three assertions were generated: *Suggestive* (relationship of perceived meaning), *Declarative* (relationship of stated facts), *Implicative/Distinctive* (relationship based on implied significance). *Suggestive* statements were often made when deriving the patient care plan from the presented symptoms/signs and/or suggesting a potential approach to acquire any missing information about the patient that was deemed necessary to have prior to assessment. *Declarative* statements often referred to the specifics of the patient care plan, proposed diagnostic approach in relation to clinical patient standards, or causal statements regarding specific child and adolescent mental health clinical practice standards. *Implicative/Distinctive* assertions were made to imply significance and were often specified to noted patient attributes readily described in the available EHR information.

**Table 3:** Think-aloud protocol analysis: referring phrase analysis

Concepts & sub-concepts(s)	Definition	Excerpt from verbal protocol
Patient/Electronic Health Record (EHR) - Available Information - Missing/Needed Information	Patient information, history, and situation or questions about unknown information regarding patient/context	“And this happens in school? Okay I would like *more information* from school *about his playmates and environmental school stressors.”*
Action	Stated method for performing an act to achieve an aim	“…for example, *there is something called the ADHD rating scale*. So, you would see if it were clinically relevant or not based on the score.”
Source	Place/person from which information was obtained/could be obtained	“I guess he would not get admitted to *the clinic [CAMHS]* with *this as the referral [from primary care]*.”
Setting	Place/environment where patient sign/symptom occurred or noted component of patient care	“It *depends on the setting* and if this is the first assessment in CAMHS…but needs to be assessed by pediatrics first…”
Sign	Objective information reported by clinician/provider that reflects patient status	“…if father and brother have an ADHD diagnosis so there may be a hereditary component which means that there may be a slightly increased risk for it.”
Symptom	Subjective information reported by patient that reflects patient status	“… [we will usually go to] when I think they have the emotional regulation problems [and then] I search to the family and see if there are any problems with *the same symptoms in the family* and thinking of if there is some heredity in the family…”
Time	Indicated chronological reference	“I would agree with the decision tree here that we *wouldn’t start with the ADHD evaluation*, but it *would be relevant later on*.”
Value	Statement of utility/importance/priority of information and proposed actions	*“*So, I would *begin* with exploring his functioning before the age of 12.”
Clinical Hypothesis - Diagnosis(es) - Related Factors	Working diagnosis/diagnoses and noted factors related to patient status	“…so, I would think more in the *general learning disability maybe when it comes to diagnosis*, not necessarily ADHD.”
Treatment	Received or proposed future treatment- environmental (educational/training, psychotherapy) or pharmacotherapy (medications and psychotherapy)	“To do a further assessment would be appropriate for her if we were going to go into doing some *treatment [for ADHD]*.”

The final step of the protocol analysis, SA, identified the following predominant reasoning and decision-making processes were involved in the patient case assessment: *Evaluate* (to review and take into consideration the available patient information); *Reflect* (to contemplate/speculate about the patient’s health status and potential care requirements based on what is known from the available EHR information); *Propose* (to conclude a hypothetical working diagnosis and/or plan for how to approach the patient’s care); and *Explain* (to provide elaboration of proposed care plan with rationale and reference to standard clinical practices).

#### Information processing models: cognitive pathway summary

**Fig. 3:**
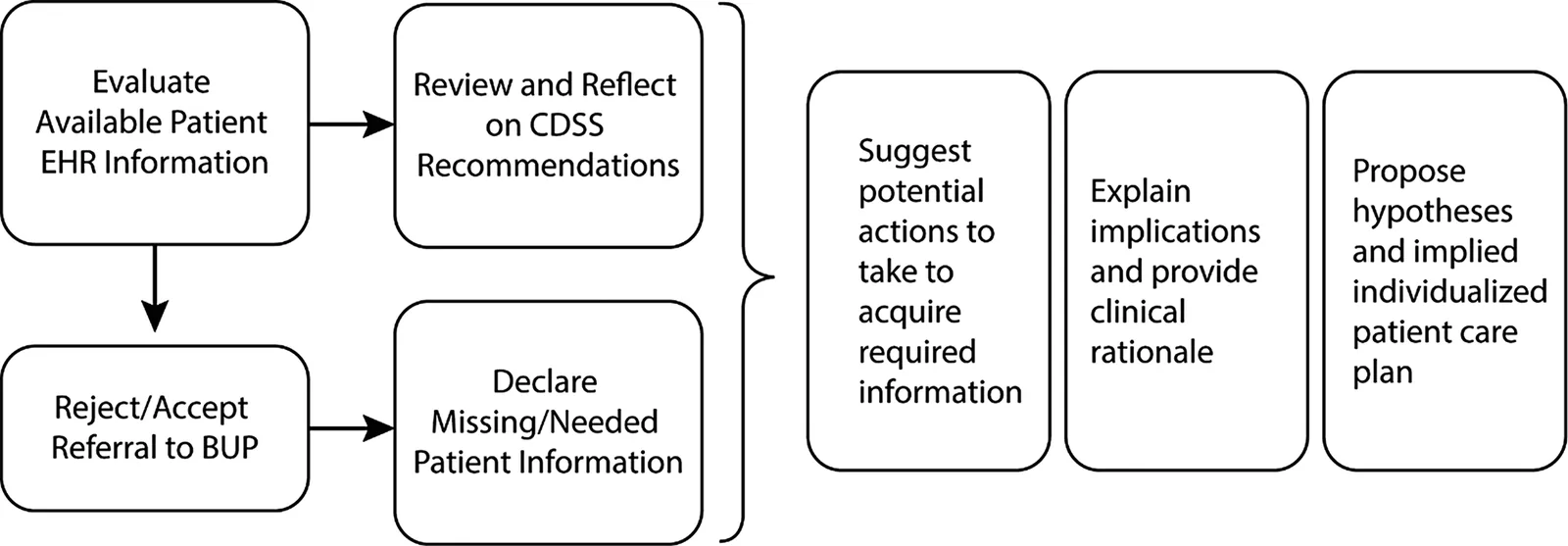
Cognitive pathway summary

There was variation in the number of references made to the specific concepts and assertions within participants’ TA sessions. The most frequently referenced RPA concepts were *Missing/needed patient information* (20%), *Available patient information* (18%), and *Action* (17%). The assertions among such concepts varied yet predominantly were *Suggestive* (49%). In conjunction with the operators extracted from SA, a summary cognitive pathway example was constructed from the compiled information processing models. The cognitive pathway example serves as a hypothetical clinical approach to assessment of a patient case referral received within CAMHS in Norway (see Fig. 3).

**Fig. 4:**
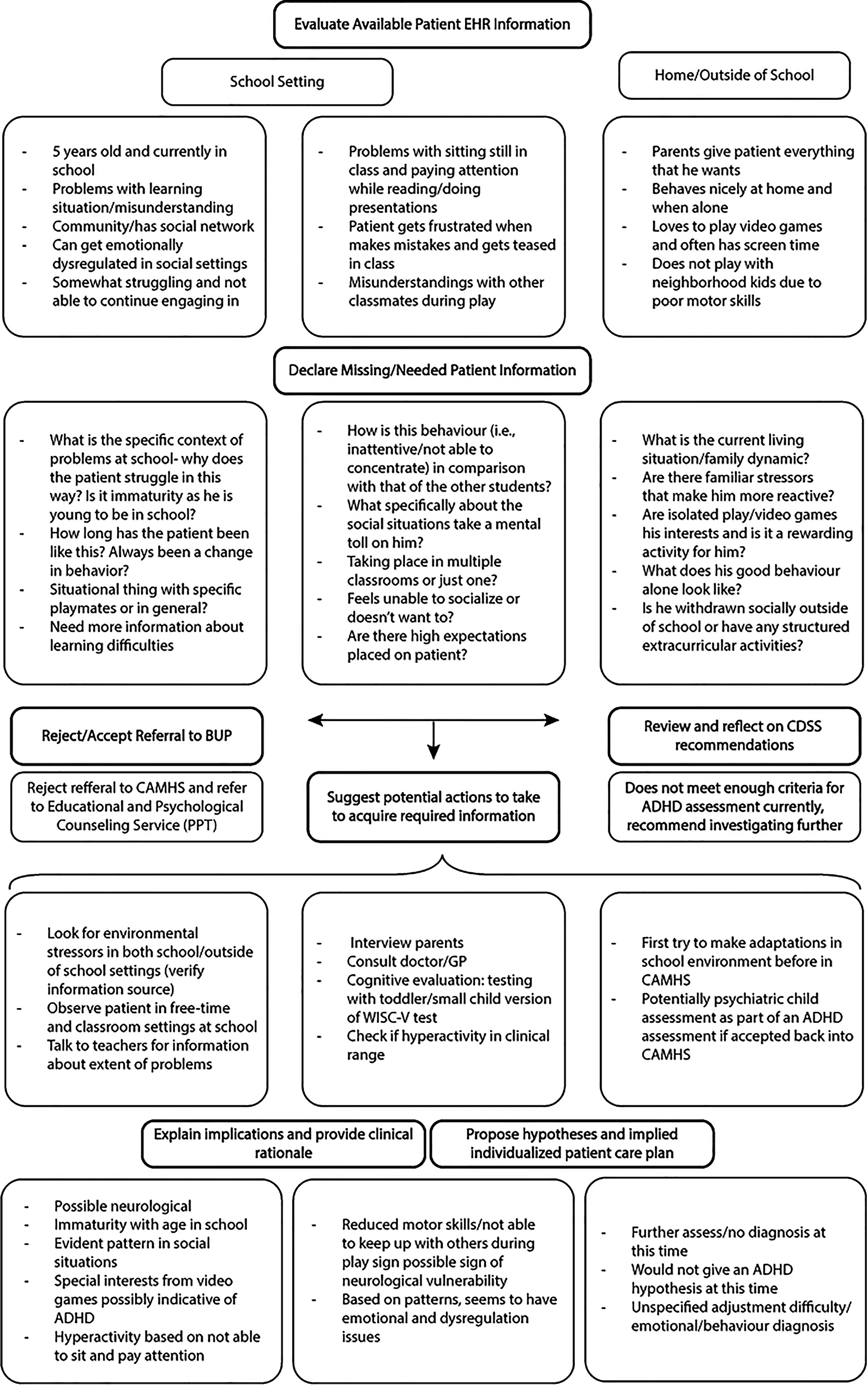
Information processing model example

The pathway followed by the CAMHS clinicians began with the participant first evaluating the available patient information. With careful consideration of that information, participants derived any suggestive relationships between stated problems, presenting symptoms/signs, and the patient’s history. All patient history information was closely analyzed to infer potential for further assessment of the patient within CAMHS, or whether receiving care from other services would be more advantageous and appropriate for the patient. This was followed by participants declaring their approach to take within CAMHS in accordance with the implications of the available medical history, family history, source of the patient’s information, settings in which the problems occur, and additional specifics to inform their assessment plan. Participants engaged with the guideline support throughout, making clear distinctions between their working hypotheses and the standard affiliated steps to take for appropriate patient assessment and potential diagnostic evaluation. From this, the implied significance of patient problems and distinctive relationships were further clarified, specifically as to what aspect of the patient situation indicated a need for further assessment and obtaining more information about the patient. After declaring the clinical practice standards that would be relevant to the specific patient case, based on accessed support and previous clinical experience, all acquired knowledge was compiled to provide final clarification of the proposed individualized patient care plan (see Fig. 4).

#### Think-aloud: usability content analysis

Based on the inductive and deductive analysis of all TA transcripts, as a result the usability thematic-content analysis revealed a total of five usability thematic categories: *Navigation*, *Workflow*, *Content*, *Usefulness*, and *Understandability.* Participants made a total of 55 comments concerning the usability of attributes of the IDDEAS 1.0 prototype. The number of comments by each participant ranged from 0 to 8. Overall, the commentary distributions across the usability categories: 15 comments (27%) concerned *Content*; 15 comments (27%) discussed *Understandability*; 14 comments (26%) addressed *Workflow;* 10 comments (18%) related to *Usefulness*; and 1 (2%) concerned *Navigation* (see Table 4).

**Table 4:** Think-aloud usability content

Usability category	Noted prototype attribute	Example comment/Text excerpt
*Navigation*		
	Guideline and patient information side-by-side when need to access	“…so this is actually quite easy to do this because its good structure on it- I have the text here and the guidelines here”
	Navigation through guideline with manual selection of criteria	“I even want to explore by clicking through to see how guideline responds to selection.”
	Simplicity of guideline checklist based on extent of information presented	“okay, so its clickable and it’s not too much information at once”
*Workflow*		
	Appropriateness of presentation of guideline content/sequence for natural clinical procedure/workflow followed	“Need to be able to assess for impulsivity and hyperactivity separately- there can be overlap in the end but should not be assessed together (does not match normal diagnostic clinical workflow) …”
	Elicits clinical reflections within clinical workflow/decision making	“Helps guide the clinician to consider why the patient might not be responding to certain treatments as expected.”
*Content*		
	Specific guideline phrasing clear alignment with standard patient assessment procedure	“So, if I click okay and then I’m also uncertain of what does this mean- “assessment examination is complete”- because it doesn’t make sense in what I have done because the assessment is not complete…”
	Automatic specification of ADHD guideline	“It is really easy to be biased with the visible ADHD guidelines on the screen.”
*Usefulness*		
	Alignment with clinical standards	“Would trust to use this in clinical work…I think that is it useful and a very good start- could use this is in my clinical work instead of standardized assessment”
	Helpfulness depends upon clinical experience of clinician	“First 3 criteria listed are not helpful because it is automatic for most clinicians, only criteria one could be helpful for some.”
	Decision tree format automatic presentation of “accept or reject recommendation”	“From what we know patient will not fulfill criteria but that does not mean she does not, just lacking information.”
	Guideline decision tree formatted into Yes/No selection requirement	“Not all patient information is either yes or no, often do not know information yet”
	Elicits reflections about potential patient care requirements	“It’s a nice way to sort of help you think through why the patient might not respond as expected…”
	Limitation of potential diagnostics exploration based on prototype provision of ADHD guideline only	“I would like to be able to see both hypotheses in parallel.”
	Access to assessment/screening tools	“So, it is helpful to a degree but if it includes assessment methods like the ADHD rating scale and the percentiles for age and sex, then that would be more helpful. We don’t do the ADHD rating scale with one informant, usually do it with parents and depending on the age of the patient, we would do it with themselves and teachers/kindergarten personnel, so you would have [on UI] different informants.”
*Understandability*		
	Guideline start prompt on user interface	“When to click “continue” to use as criteria to check for the specific patient”
	Automatic presentation of guideline and expectation of inability to use guideline-based recommendations	“Too little information for any assessment using a guideline- if this would be a referral, we might even turn it around and send it back.”
	Presentation of generated guideline recommendation	“…and this is a bit confusing (referring to first guideline box), is this my recommendation or my answer? This may cause real concern because I don’t know how to interpret it.”

The sentiment analysis revealed that all statements concerning the usability of specific prototype attributes were made with either “critical” or “positive” sentiment, with no statements made with neutral sentiment. Out of the 55 comments made, 49 (89%) had a critical sentiment, while 6/55 comments (11%) had a positive sentiment. With regards to the USI cycle stages, 4/55 (7%) of the comments occurred within the Planning stage, 4/55 (7%) in the Translation stage, 22/55 (40%) in the Physical Action stage, and 25/55 (46%) in the Assessment stage. From the negative sentiment usability comments, participants specified 12 problems and proposed 17 adjustments to the IDDEAS prototype. Of the 12 problems, three had more than one affiliated adjustment proposed (see Table 5).

**Table 5:** Noted prototype attributes/problems encountered and proposed adjustments

Specified problem encountered	Proposed adjustment
System automatically provides support with IDDEAS guidelines	The system provides confirmation to clinicians that guideline support is up to date with the current standardized clinical guidelines used in local practice
Automatic prompt to continue into the guideline on start screen is distracting for planning assessment	Do not present guideline on user interface “home screen”
Presentation of specified guideline (i.e., ADHD guideline) presents possible bias	Leave for clinicians to choose which guideline to use
Guideline automatically presented creates feeling of obligation to use for patient case assessment (i.e., reject referral) even if clinician deems not relevant for assessment at that time based on available patient information	- Identifies when potentially too little information/if referral should be rejected - Include list of different informants - Provide place to indicate who provided information (or any completed rating scales)
Forced to navigate through guideline decision tree at early stage of patient assessment	Need to be able to select criteria based on patient information in dynamic way, not required to go through yes or no criteria and have option to select what is “known” and what is “unknown” patient information
Unclear terminology in guideline support recommendation box	Recommend using different terminology specific to the stage of the overall patient assessment (i.e., screening assessment versus complete patient assessment)
Narrow focus with guideline content criteria built on if/then does not provide option for open investigation or parallel assessments for overlapping symptoms	Make it more dynamic for specialists with the provision of both the met criteria and unmet criteria, side by side (i.e., for hyperactivity/impulsivity separately)
Yes/No requirement forces decisions with inability to identify unknown information	- There should be an “I do not know” option, rather than forcing selection of Yes or No. - Provide alert to indicate that clinician does not know specific information & presentation of summary of unknown patient information
Recommendations are not customized and do not adapt in real time to the individual patient information	Integration with patient EHR and make system dynamic so can fill in with patient information as acquire more
Limitation of guidelines to ADHD does not allow for full exploration of symptoms	Include all standardized clinical guidelines to allow for unstructured processes before narrowing down and be more applicable for complicated evaluations
Does not prompt clinicians to access the assessment tools that would want to consider/complete for the patient assessment	- Clinicians receive an alert when there are specific screening tools/rating scales the system recommends that the clinician use for the patient and are provided with direct access to the specified materials - When the measurements are completed, the system provides the clinician with the patient’s rating scale score and coded patient score distributions to allow for comparison with population data - The system will present the results from the patient’s assessment measures with population percentiles to show where the patient falls in comparison to the normal for gender/age
Content potentially redundant for expert clinicians	Provide the guideline recommendations in checklist type format (i.e., decision tree) in the primary care services (i.e., first point of care) before referral is made to CAMHS

## Discussion

The main findings of the study can be summarized as follows: clinicians in CAMHS appreciated the simplicity, display and functional ease-of-use, usability, and engagement of IDDEAS prototype 1.0 content. Additionally, the UCD methods provided critical insight into clinicians’ clinical decision-making (CDM) processes, to help with optimizing future seamless integration of the system that can provide helpful support while also maintaining clinician autonomy. While clinicians found specific aspects of both the UI display, and functionality of the prototype to be advantageous and potentially helpful at the point of care, they also indicated that the system requires further development before it can serve as a useful tool in their practices. Overall, clinicians expressed interest in a more developed system that can provide timely evidence-based decision-making support, with recommendations adhering to clinical standards, adapting to the individual patient, and ultimately coinciding with their clinical workflows.

### Decision support appropriateness and workflow alignment

At the start of the TA sessions, participants questioned the clinical appropriateness and relevance of IDDEAS. During the planning stage of the USI cycle, participants consistently clarified whether the available patient information presented in the vignette justified an accepted referral to CAMHS or not, before providing their critical rationale behind the initial assessment planning. To address this, clinicians recommended that the next version of IDDEAS first provide them with an alert to consider rejecting a patient referral, or an option to request additional patient information. Also, the system suggests a specific clinical guideline to be considered, but only after the clinician has completed an initial planning stage and started the assessment. Other CDSS development studies have found that clinicians are inclined to first acquire additional information rather than potentially overlooking any red flags, as well as to receive a trigger from the tool only after the clinician has first independently gone through the patient’s health history [110, 115, 116]. One of the most important considerations for ensuring the development of a useful CDSS is the assurance that clinicians will maintain control of decision-making, and their autonomy will be respected [55, 61, 117].

The simulated assessment of a hypothetical case scenario prompted clinicians to provide their authentic reflections on the current version of the IDDEAS system and proved insightful as to both their natural cognitive processing and approaches to CDM. The compilation of the common cognitive pathway revealed that participant information processing within their simulated workflow approaches consistently aligned with standardized clinical diagnostic practices. While this could potentially be considered related to the prevalence of ADHD in CAMHS [10, 11], and that clinicians are quite experienced with diagnosing it [78–80], participants consistently did not rush to reach a conclusion but rather thoroughly evaluated all components of the patient scenario and provided rationale for their clinical processing. The main concepts and reasoning processes used in the TA protocol demonstrated that their CDM is thoroughly analytical, based on pattern recognition, and attuned to identifying gaps in the available information, regardless of the patient case complexity. The compilation of the information processing models into the pathway uncovered the most common CDM components and critical approaches to information synthesis, informing the epistemological base for the system’s data-based recommendations and reiterating the potential for clinicians to avoid an obligatory adherence to CDSS-generated advice over their own critical assessment when using at the point of care.

### Prioritization of clinician autonomy and system adaptability

While other studies have found that lack of clinician adherence to guidelines could pose risks for diagnostic errors and bias [63, 110], in our study the clinicians themselves raised a concern about the risk of a biased assessment. Not only did their CDM align with the guidelines, with a consistent hesitation to assume a hypothetical diagnosis, automatic presentation of a specific guideline on the UI home-screen (e.g., ADHD), was noted as potentially problematic, due to the possibility of biasing their assessment. While the majority (72%) of participants agreed that it is helpful to have patient information displayed directly next to the guidelines, they suggested it might be more advantageous to allow clinicians to select the ADHD guidelines in the module, rather than having it presented automatically. Several clinicians noted that the future version of IDDEAS, providing access to all standardized clinical guidelines, and allowing the clinician to choose when and which specific guideline to use, could help overcome the possibility of a biased assessment. While over-confidence bias can prove risky to healthcare in general [110, 118, 119], it is also important that UCD methods allow for the clear prioritization of clinician autonomy and their role as decision makers. A fully developed CDSS needs to show trust in the clinicians’ CDM [70, 120].

The participants distinctly pointed out that the content reflected CAMHS standardized practices and the layout of criteria was clinically appropriate, matching their intuitive clinical assessment process. However, while participants noted that the “clickable” format was helpful, with not too much information provided at once, they also raised a concern that the content could potentially be redundant for expert clinicians. Other CDSS studies have also found that clinicians believed the suggested guidelines were at times hindering their CDM – when the content did not seem necessary for expert clinicians [121], appropriate to the specific patient, or reflective of valid clinical practices [115, 116, 122]. With 11 separate mentions, being required to navigate through the entire guideline decision tree was the most frequently noted problem. Even so, this input also came with the suggestion that the decision tree/checklist formatting would potentially optimally suit novice clinicians well and could even assist general practitioners (GPs) in making their referrals to CAMHS. While this has yet to be explored, the expansion of a CDSS for primary care services could prove valuable to the cross-sectoral, collaborative nature of patient care by strengthening shared decision-making across the services [58, 123], potentially efficiently expanding the capacity of CAMHS through enhanced primary care competencies in specialized child and adolescent psychiatry (CAP) [124, 125].

Participants emphasized that guideline support should be both context-sensitive and flexible, aligning with the specific stage of the patient assessment and the information currently available in the EHR. To address potential gaps in patient data, they recommended that IDDEAS include an option to have the system mark diagnostic criteria as “Known” or “Unknown,” reducing unnecessary time spent navigating incomplete records. Additionally, participants felt that requiring the selection of “Yes” or “No” also hindered their investigation and suggested adding an “I do not know” option. CDSS alerts and recommendations need to be clear and understandable, to avoid unnecessary confusion for clinicians who already face information overload [33, 70, 112]. One study found that an effective CDSS may need to be passive rather than active, with triggers timed appropriately around decision-making, not abruptly interrupting the process [110]. The discrepancy between CDS and a clinician’s natural clinical workflow can be an issue if the support is not consistent with their cognitive processing of information [33, 70, 73].

### Integration and transparency for trustworthy support

Participants also noted transparency and access to evidence as key components of the usability of IDDEAS. When asked if it would be helpful to have a summary of why the assessed criteria are met or not, 48% of participants strongly agreed, with evidence presented as population-based percentile data, or similar patient case examples. This coincides with a desire for more transparent and explainable decision support within the CDSS [120], with clinicians showing interest in receiving explanations for recommendations or links to reference materials for recommendations [61]. Participants also proposed that the updated system should automatically generate a list of professionals to contact and sources from which the information has been acquired, suggest assessment measures or scales to use, and generate an overview of which diagnostic assessment criteria are fulfilled as new patient information is obtained. All adjustments will require close integration with the EHR, which will ultimately allow for dynamic assessments that are up-to-date with each individual patient’s status as well as the current standards.

In both the IDDEAS interview sessions, and the TA sessions, participants shared their hope that IDDEAS will be integrated with the patient health record, for optimal decision support [77]. Integrating such a tool with EHR allows for the use of advanced data analytics, such as natural language processing (NLP) techniques, to detect changes in the EHR information and extract relevant data to enhance personalized decision support [33, 126]. Integration with the local EHR system is an important component of CDSS development and is essential for successful implementation, specifically for the transportability and interoperability of the system [13, 34, 111, 121, 127]. Clinicians want decision support that is suitable to a variety of complex cases, while also adaptable to be appropriate for each individual patient to receive personalized care [120].

### Strengths & limitations

Since this study served as a formative usability evaluation of a low-fidelity prototype, it offers important insights for further usability research. While considered advantageous for measuring usability in depth, methods for direct measurement of quality of decisions, work speed, improved performance during use, and other features (e.g. eye-tracking), were determined to be difficult to control outside the setting of a usability lab.

The SUS and UES were modified and translated for use in the IDDEAS study context, and based on CFA findings, their application should be limited to this setting. For instance, while the overall SUS score indicates adequate internal consistency according to established scoring standards, the RMSEA value for the IDDEAS adapted SUS falls slightly outside acceptable thresholds [97, 101, 128]. One possible explanation for the suboptimal model fit is the limited sample size [129], with the ratio of participants to questionnaire items possibly having an effect, depending on the factor analysis [88, 93, 95]. However, for the UES, CFA is recommended over Exploratory Factor Analysis (EFA), or principal components analysis (PCA) [88], and given the exploratory and multifaceted nature of this study, increasing the sample size was not feasible. Despite these limitations, the initial CFA results provide valuable insights into the reliability of the scales following translation and cultural adaptation.

There is a potential for bias, as the convenience sample may disproportionately represent clinicians who are already knowledgeable about CDSSs or who have a particular interest in innovative interventions for CAMHS. Also, while the TA method provided valuable insight into CDM and information processing, it is not an intuitive process, and some participants might have found it off-putting to think aloud through one’s thought process while interacting with the system. This subjective degree of comfortability may have contributed to the substantial variation in the duration of the TA sessions amongst the participants, as well as the amount of information reflected upon. However, unlike the retrospective think-aloud method, the concurrent think-aloud method avoids prompting and focuses on participants’ natural verbalizations within CDM, which allows for authentic real-time reflections [83, 116]. By completing the think-aloud session concurrently with the simulated patient case scenario, clinicians were able to describe their approaches in an unfiltered manner. This brought a greater understanding of how decision support could fit with their decision-making and current clinical practices, and allowed for authentic reflections about IDDEAS 1.0 attributes, and how a fully developed IDDEAS could be useful in CAMHS.

To the best of our knowledge, this study is the first to use a simulated scenario approach to assess the current usability of a CDSS prototype for CAMHS in Norway. This study provides a CDSS UCD and formative usability testing framework, as well as actionable insights to enable collaborative co-design and development, to realize the full potential of CDSS for CAMHS in the future. Future research for the development of IDDEAS should involve “near live” usability evaluations of the updated system that utilize patient actors or videos, rather than hypothetical patient case scenarios [61, 110]. IDDEAS prototype 1.0 should be updated in line with the results of this study, adhering to the proposed adjustments and feedback received, as well as incorporating the data-driven component to complement the guideline-based support. The evaluation of the IDDEAS prototype 2.0 should be conducted in iterations, to allow for comparison with a prompt development turnover [130, 131].

## Conclusion

The scenario-based exploration of the IDDEAS prototype allowed CAMHS clinicians to provide input about the decision support system they have available in their clinical work, and what they wish for in the future. Clinicians were able to suggest innovative solutions to meet any of these needs, and how future system development will help them provide mental health care for children and adolescents in Norway. This study has produced a better understanding of how clinicians in CAMHS interact with IDDEAS as a CDSS prototype for ADHD diagnosis and treatment, their cognitive pathways, their approach to information processing, and how their proposed IDDEAS adjustments will best support clinicians in real time, seamlessly, at the point of care.

## Appendix I

Confirmatory factor analysis of a one-factor model of IDDEAS-SUS for the *n* = 63 participants. Standardized factor loadings and correlations.

**Figure:**
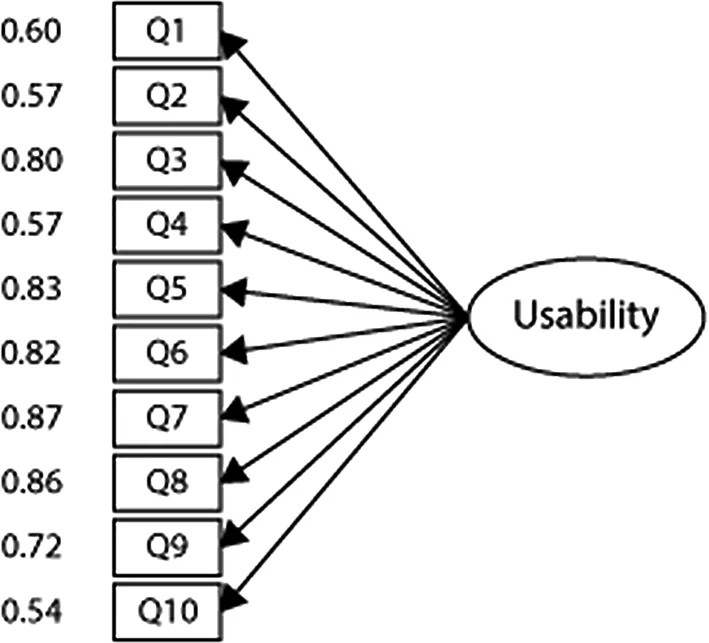

Confirmatory factor analysis of a two-factor model of IDDEAS-SUS for the *n* = 63 participants. Standardized factor loadings and correlations.

**Figure:**
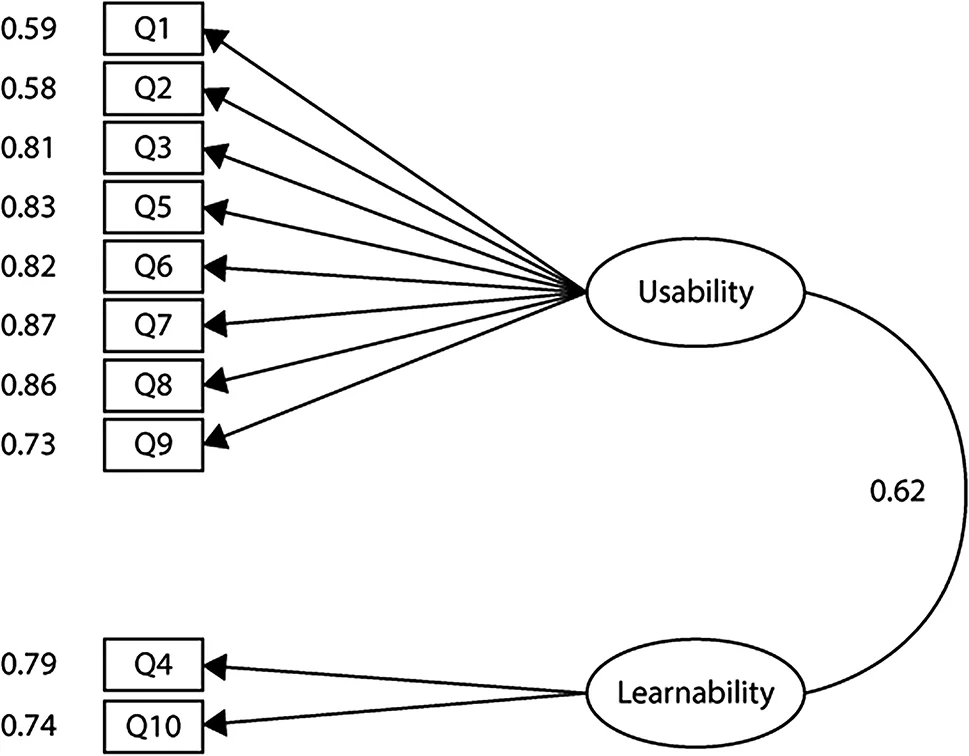

## Appendix II

Confirmatory factor analysis of a four-factor model of IDDEAS-UES for the *n* = 64 participants. Standardized factor loadings and correlations.

**Figure:**
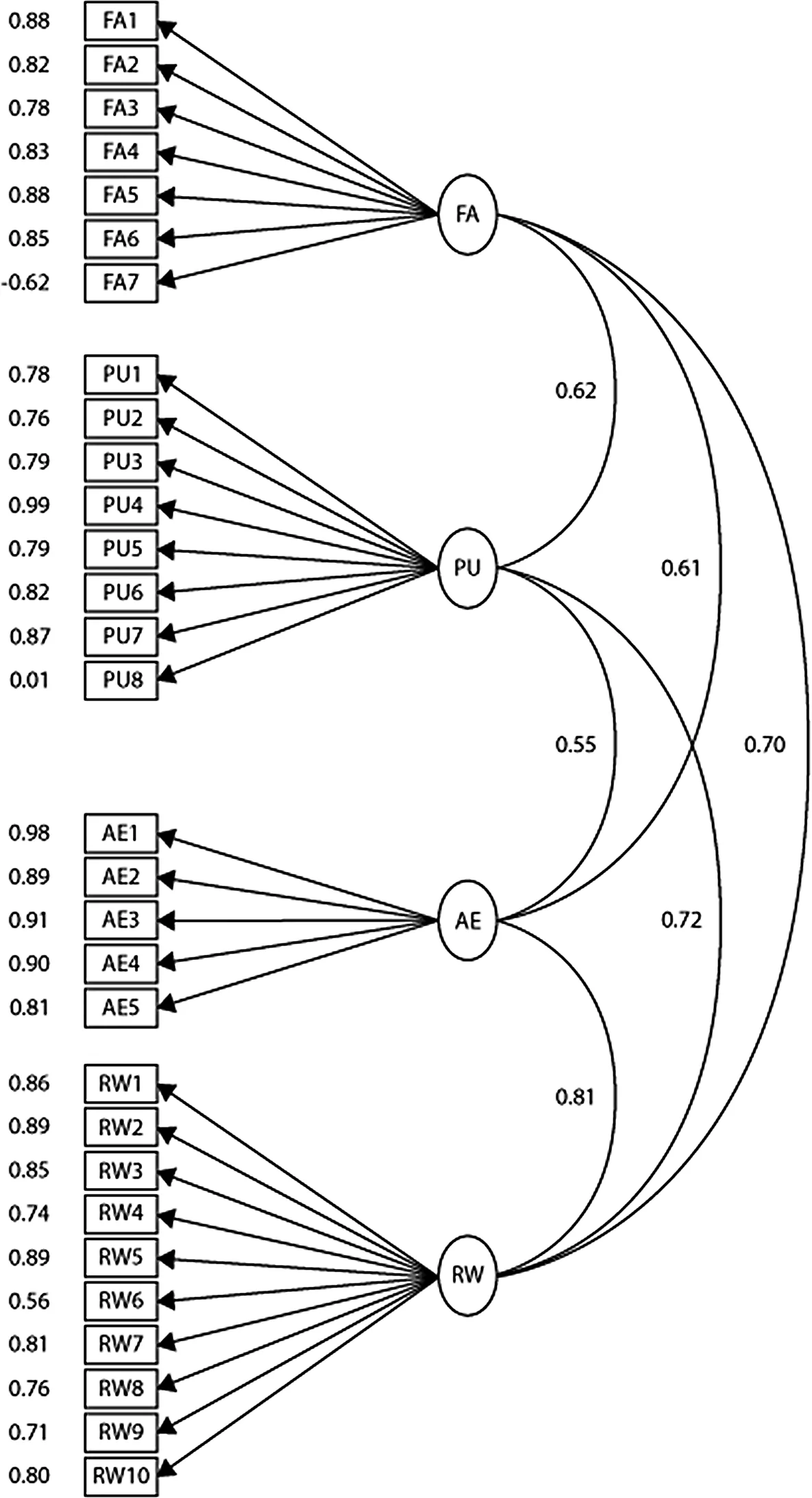

## Supplementary Information

Below is the link to the electronic supplementary material.

Supplementary Material 1

## Data Availability

The dataset used and/or analyzed during the current study are available from the corresponding author on reasonable request.

## Abbreviations

AA: Assertion analysis
ADHD: Attention-deficit/hyperactivity disorder
AE: Aesthetic appeal
AI: Artificial intelligence
BUP: Barne og Ungdomspsykiatrisk (CAP in Norwegian)
camhs: Child and adolescent mental health services
CAP: Child and adolescent psychiatry
CDSS: Clinical decision support system
CDM: Clinical decision-making
CFA: Confirmatory factor analysis
CFI: Comparative fit index
CTA: Concurrent think-aloud
DSM-V: Diagnostic and statistical manual of mental disorders, 5 ed
HER: Electronic health record
EFA: Exploratory factor analysis
FA: Focused attention
GP: General practitioner
HCI: Human computer interaction
HIT: Health information technology
HIS: Human system interaction
ICD-10: International classification of disease, 10th revision
ICM: Integrated care model
ICT: Information and communication technology
IDDEAS: Individualized digital decision assist system
ML: Machine learning
NPR: National patient registry
N-BUP: Norwegian association for child and adolescent mental health
NLP: Natural language processing
PCA: Principle components analysis
PU: Perceived usability
RMSEA: Root mean square error of approximation
RPA: Referring phrase analysis
RW: Reward
SA: Script analysis
SPSS: Statistical package for social sciences
SUS: System usability scale
TA: Think-aloud
TLI: Tucker Lewis index
UCD: User-centered design
UES: User engagement scale
UI: User-interface
USI: User-system interaction
UX: User-experience
WHO: World Health Organization
WLSMV: Weighted least square mean and variance
XAI: Explainable artificial intelligence
