# Supplementary material for: Clinical decision support system for child and adolescent mental health services: a formative usability study
Source: BMC Med Inform Decis Mak. 2026 Jul 20;26:414. doi: 10.1186/s12911-026-03720-w (PMC13670641; doi:10.1186/s12911-026-03720-w)
Supplement: Supplementary file 1 [file 12911_2026_3720_MOESM1_ESM.pdf]

IDDEAS Display and Functional Ease-of-Use Questionnaire

Survey Item	Strongly disagree	Disagree	Do not disagree or agree (Neutral)	Agree	Strongly agree
1. The interface navigation buttons simplify the process.					
2. It is helpful to have the patient information displayed next to step-by-step guidelines.					
3. Provision of guidance in step-by-step stages is helpful.					
4. Displaying error notifications in red text on the screen is helpful.					
5. It is useful to have a back button readily available.					
6. The terminology used in the program is understandable and appropriate.					
7. It was not easy to go back and correct any mistakes made while following the guidance steps.					
8. The system is designed in a way that will be easy to use for clinicians with different skills and experience.					
9. It should be possible to look up patient information.					
10. It would be helpful to have a summary of why the assessment criteria was met or not met.					
